# ALK-negative retroperitoneal inflammatory myofibroblastic tumor treated conservatively: case report

**DOI:** 10.1093/jscr/rjaf709

**Published:** 2025-09-05

**Authors:** Fu-Xiang Lin, Wei Wei, Yi Yu, Yuan Mai, Le Xie, Zhan-Ping Xu

**Affiliations:** Department of Urology, The Eighth Clinical Medical College of Guangzhou University of Chinese Medicine, No. 6 of Qinren Road, Foshan 528000, Guangdong, People’s Republic of China; Department of Urology, Foshan Hospital of Traditional Chinese Medicine, No. 6 of Qinren Road, Foshan 528000, Guangdong, People’s Republic of China; Department of Urology, The Eighth Clinical Medical College of Guangzhou University of Chinese Medicine, No. 6 of Qinren Road, Foshan 528000, Guangdong, People’s Republic of China; Department of Urology, Foshan Hospital of Traditional Chinese Medicine, No. 6 of Qinren Road, Foshan 528000, Guangdong, People’s Republic of China; Department of Urology, The Eighth Clinical Medical College of Guangzhou University of Chinese Medicine, No. 6 of Qinren Road, Foshan 528000, Guangdong, People’s Republic of China; Department of Urology, Foshan Hospital of Traditional Chinese Medicine, No. 6 of Qinren Road, Foshan 528000, Guangdong, People’s Republic of China; Department of Urology, The Eighth Clinical Medical College of Guangzhou University of Chinese Medicine, No. 6 of Qinren Road, Foshan 528000, Guangdong, People’s Republic of China; Department of Urology, Foshan Hospital of Traditional Chinese Medicine, No. 6 of Qinren Road, Foshan 528000, Guangdong, People’s Republic of China; Department of Urology, The Eighth Clinical Medical College of Guangzhou University of Chinese Medicine, No. 6 of Qinren Road, Foshan 528000, Guangdong, People’s Republic of China; Department of Urology, Foshan Hospital of Traditional Chinese Medicine, No. 6 of Qinren Road, Foshan 528000, Guangdong, People’s Republic of China; Department of Urology, The Eighth Clinical Medical College of Guangzhou University of Chinese Medicine, No. 6 of Qinren Road, Foshan 528000, Guangdong, People’s Republic of China; Department of Urology, Foshan Hospital of Traditional Chinese Medicine, No. 6 of Qinren Road, Foshan 528000, Guangdong, People’s Republic of China

**Keywords:** inflammatory myofibroblastic tumor (IMT), retroperitoneum, ALK-negative, corticosteroids, ureteral obstruction

## Abstract

A 55-year-old female presented with left flank pain and ureteral obstruction. Imaging revealed a retroperitoneal mass suspicious for malignancy. Histopathology confirmed an inflammatory myofibroblastic tumor (IMT; anaplastic lymphoma kinase [ALK]-negative, mouse double minute 2 homolog-positive). Due to vascular encasement prohibiting safe resection, cystoscopic ureteral stenting was performed followed by 12 months of prednisone and celecoxib therapy. Serial imaging showed progressive regression, with near-resolution at 36 months. This demonstrates successful medical management of unresectable ALK-negative retroperitoneal IMT.

## Introduction

Inflammatory myofibroblastic tumors (IMTs) are intermediate-grade mesenchymal neoplasms rarely involving the retroperitoneum [1, 2]. These lesions frequently mimic malignancies, causing diagnostic uncertainty. While complete resection remains standard, we document conservative management of an unresectable anaplastic lymphoma kinase (ALK)-negative retroperitoneal IMT.

## Case report

**Figure 1:**
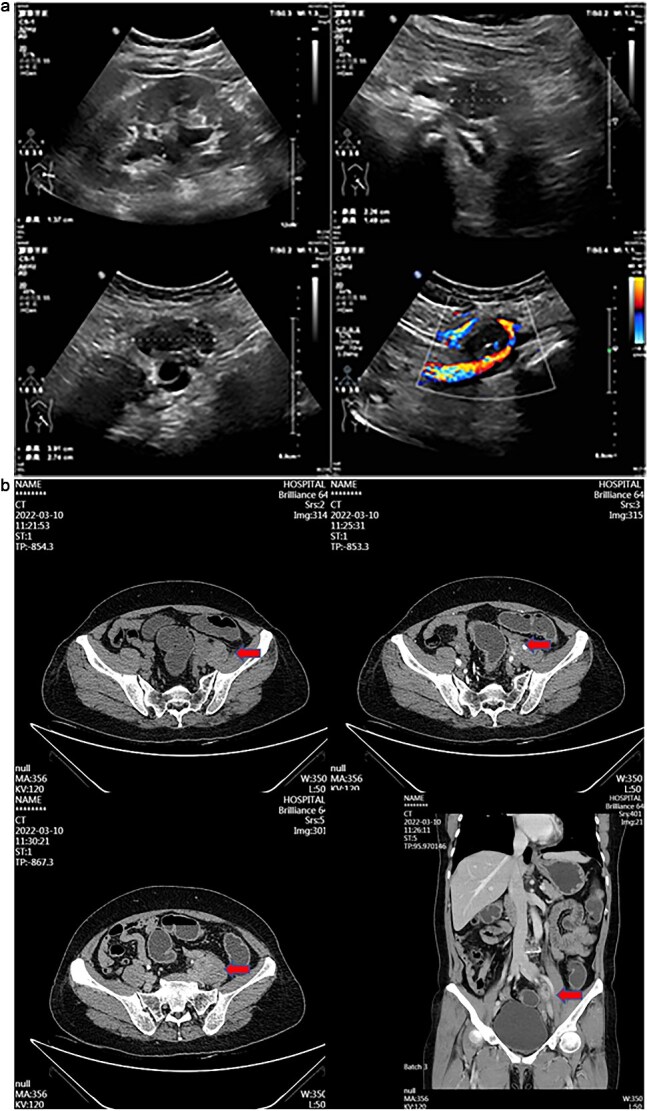
(a) Transabdominal ultrasound image showing hypoechoic and mixed-echoic masses adjacent to left iliac vessels with associated mild left Hydronephrosis and mid-ureteral compression. (b) Contrast-enhanced CT image demonstrating ring-enhancing left adnexal mass and left iliac vessel nodule with associated ureteral compression and mild hydroureteronephrosis.

A 55-year-old female patient has experienced persistent pain in her left lower back for more than 3 weeks. Ultrasound examination revealed a hypoechoic mass and a mixed echoic mass beside the left iliac vessel, the nature of which was undetermined. There was a small amount of effusion in the left kidney. It was considered possible that the middle section of the left ureter was compressed (Fig. 1a). Computed tomography (CT) examination showed a nodular soft tissue density shadow was observed beside the left iliac vessel, ~17 × 20 × 30 mm in size. After enhancement, it showed annular enhancement. The lesion was slightly compressed adjacent to the left ureter, and above this level, the left ureter and the left renal pelvis and calyces were slightly dilated. This suggests that a nodular soft tissue density shadow beside the left iliac vessel is of undetermined nature, and the possibility of metastasis is high (Fig. 1b).

**Figure 2:**
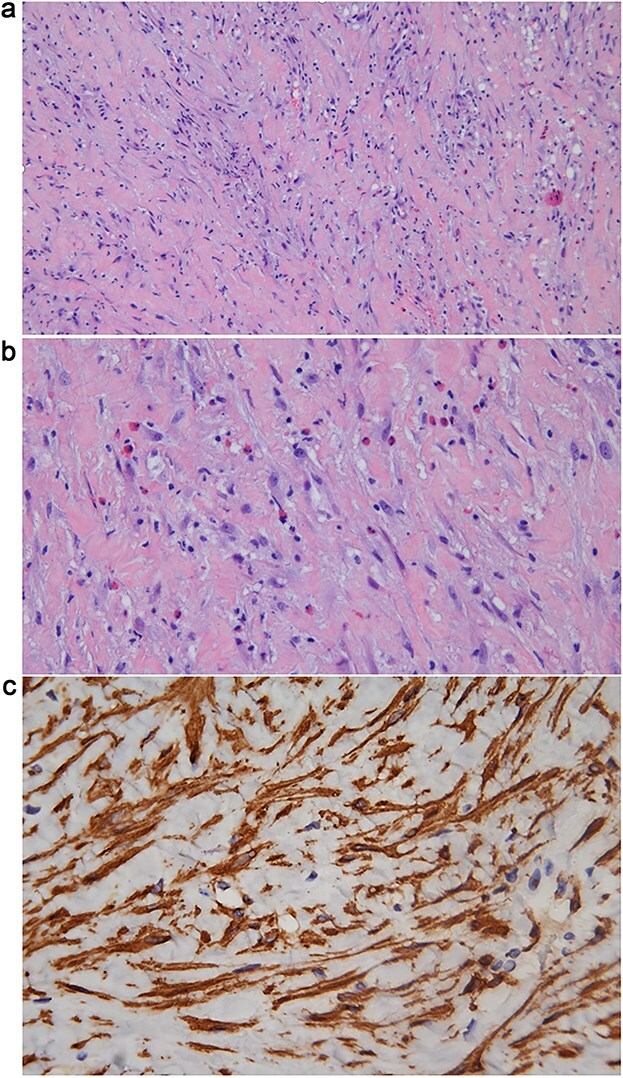
(a) Hematoxylin & Eosin (H&E) stain (10 × 10) showing proliferation of myofibroblasts with infiltration of eosinophils and lymphocytes, and intercellular collagen deposition. (b) H&E stain (10 × 20) showing proliferation of myofibroblasts with infiltration of eosinophils and lymphocytes, and intercellular collagen deposition. (c) Immunohistochemical staining for SMA (10 × 40) demonstrating cytoplasmic positivity in the proliferative spindle tumor cells.

Intraoperatively, under cystoscopy in lithotomy position, a ureteral stent was first placed. Then, the patient was repositioned to supine with legs apart in a Trendelenburg position for laparoscopy. Laparoscopic exploration revealed that the tumor encased the left iliac vessels, precluding safe resection; therefore, only a biopsy was performed. Pathological biopsy revealed spindle cell proliferation with plasma cell infiltration. Immunohistochemistry (using standard techniques with appropriate positive and negative controls) demonstrated positivity for smooth muscle actin (SMA) and muscle-specific actin, negativity for ALK, and overexpression of MDM2 (mouse double minute 2 homolog) (Fig. 2a–c).

Given 360° encasement of the left iliac vessels, radical resection was abandoned. Ureteral stenting relieved obstruction. Multidisciplinary consensus recommended medical therapy with prednisone (20 mg daily) [3] and celecoxib (200 mg daily) [4].

Serial monitoring documented:

Month 3: 60% volume reduction.

Month 12: 4 mm residual mass (ureteral stent removed at 10 months postoperatively).

**Figure 3:**
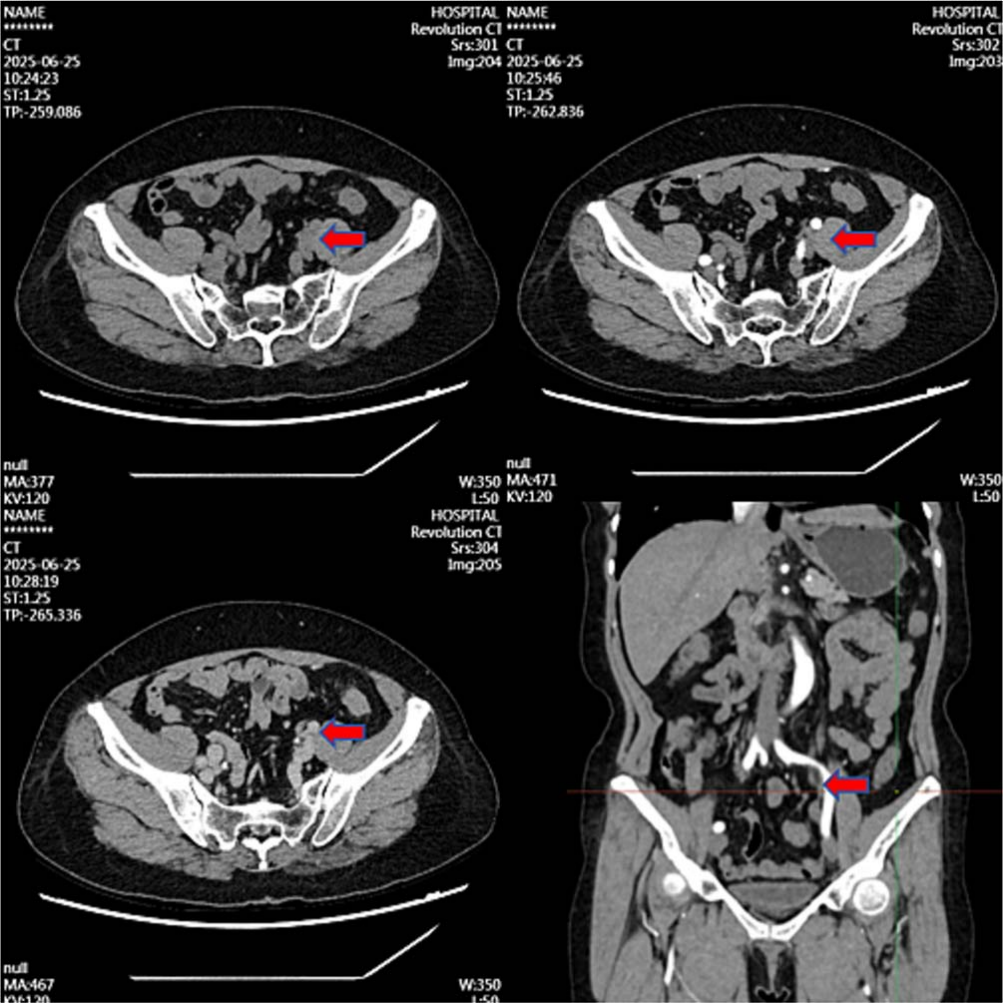
Contrast-enhanced CT image at 36-month follow-up demonstrating near-complete resolution of previously described left iliac vessel mass following medical therapy.

Month 36: Near-complete resolution (Fig. 3).

Treatment concluded at 12 months. The patient remains recurrence-free 24 months post-therapy.

## Discussion

This case presents three critical considerations:

*Diagnostic challenges:* Retroperitoneal IMTs frequently mimic malignancies radiographically. MDM2 co-expression in our case suggested sarcoma initially, necessitating ALK-FISH confirmation.

*Therapeutic implications*: Unresectable ALK-negative IMTs lack standardized protocols. Liu *et al*. reported similar responses to steroid/NSAID regimens (68% disease control) [5]. Our sustained remission without surgery expands this evidence.

*Surgical constraints*: Vascular involvement precludes complete resection in 31% of retroperitoneal IMTs [2]. Medical management avoided high-risk dissection at the pelvic brim.

Prevention of retained materials requires meticulous surgical counts. For retroperitoneal masses, early biopsy remains essential for appropriate management.

## Conclusion

Prednisone/celecoxib therapy achieved durable remission in this unresectable ALK-negative retroperitoneal IMT. This approach offers a viable alternative when resection risks outweigh benefits.
